# Understanding transitions in farming systems and their effects on livestock rearing and smallholder livelihoods in Telangana, India

**DOI:** 10.1007/s13280-021-01523-z

**Published:** 2021-03-08

**Authors:** Bhavana Rao Kuchimanchi, Imke J. M. De Boer, Raimon Ripoll-Bosch, Simon J. Oosting

**Affiliations:** 1 Animal Production Systems Group, Wageningen University & Research, P.O. Box 338, 6700 AH Wageningen, The Netherlands; 2 Foundation for Ecological Security, Jahagir Pura Road, Hadgood, Anand, Gujarat 388110 India

**Keywords:** Caste groups, Cropping patterns, Dryland regions, Intensification, Land use change, Water resources

## Abstract

Increasing food demands are causing rapid transitions in farming systems, often involving intensified land and resource use. While transitioning has benefits regarding poverty alleviation and food outputs, it also causes environmental and social issues over time. This study aims to understand the transitions in farming systems in a region in Telangana, from 1997 to 2015, and their effect on livestock rearing and smallholder livelihoods. We also examine the impact of the transitions on lower caste groups and women in particular. We collected data using a combination of methods, i.e., a household survey, focus group discussions, and secondary data sources, to build a comprehensive picture of the transitions in the region. We found that subsistence mixed farming systems transitioned to market-orientated specialized systems over a short time span. As the transition process gained momentum, households either intensified their production or got marginalized. Technological interventions, development programs with integrated approaches, and market demand for certain agricultural produce triggered increased regional production but also led to the scarcity of water, land, and labor. The transitions marginalized some of the households, changed the role of livestock in farming, and have been inclusive of both lower caste groups and women in terms of increased ownership of large ruminants and access to technologies. However, for women specifically, further increase in workload in the context of farming is also found.

## Introduction

Many developing countries have policies to transition from subsistence farming systems into market-oriented systems in response to the increased demand for animal source food. This transition is often associated with the processes of specialization and intensification of farming systems, as well as increased use of resources, such as biomass, land, and water (Tarawali et al. 2011; Alexandratos and Bruinsma 2012; Bharucha et al. 2014). While such transitions have benefits in terms of increased food output, it may also cause environmental issues, e.g., overexploitation of natural resources, and social issues such as farmer dependency on external inputs and marginalization of communities (Lebacq et al. 2013; Clay et al. 2020).

Agroecosystems in dryland areas, which are predominant regions in developing countries, face harsh agro-climatic conditions and scarce infrastructure and support services, and host diverse farming practices (i.e., pastoral, agropastoral, rainfed, and irrigated crop production). These regions are also hotspots for land degradation, low crop yields, and poverty (van Ginkel et al. 2013; Chander et al. 2014). In India, 69% of the territory is classified as dryland. To develop these regions socio-economically, several development initiatives have been implemented among which integrated watershed development programs (WDPs) have been and continued to be the forefront strategy (GoI 2008; Smyle et al. 2014). WPDs have resulted in the modernization of farming systems in dryland areas. This meant that traditional mixed crop-livestock farming systems, using local livestock breeds and crops, generally transitioned to more intensive market-oriented and specialized farming systems, using imported breeds and new crop varieties (Puskur et al. 2004; van Ginkel et al. 2013; Tian et al. 2014; Amjath-Babu and Kaechele 2015; Behera et al. 2016; Gathorne-Hardy 2016). While the transitions in farming systems have increased overall agricultural output (Rao 2000; Government of India 2006; Jhoshi et al. 2008; Wani et al 2008; Palanisami and Kumar 2009), some unfavorable side effects such as the exclusion and marginalization of some social groups (Puskur et al. 2004; Pingali 2012; Kannan 2015), increased workload on women (van Ginkel et al. 2013), and overuse of natural resources (Batchelor et al. 2003; Bharucha et al. 2014) have also been reported.

Development programs like WDPs, which have integrated approaches, are dynamic and known to trigger rapid changes in farming systems that can involve trade-offs and need to be understood further (van Ginkel et al. 2013; Reddy and Syme 2015). Moreover, research on transitions in farming systems is largely focused on farm-level studies (Robinson et al. 2015; Bui et al. 2016; Gaitán-Cremaschi et al. 2019). Regional studies of transitions by Dorward (2013), Jayne et al. (2014), Pretty and Bharucha (2014), and DiCarlo et al. (2018) reported how farming systems developed, how they interact during the transitions, and how the transition affected natural resource use. To our knowledge, there are no scientific publications about regional aspects of transitions in India.

Hence, the aim of this study was to understand the transitions in farming systems in a region in Telangana from 1997 to 2015 that has witnessed over three decades of several development initiatives including WDPs. We look closely at how transitions have occurred and their effect on livestock rearing and smallholder farming systems. We also look at the impact of transitions on different caste groups and women in particular.

## Materials and materials

### Study location

**Fig. 1:**
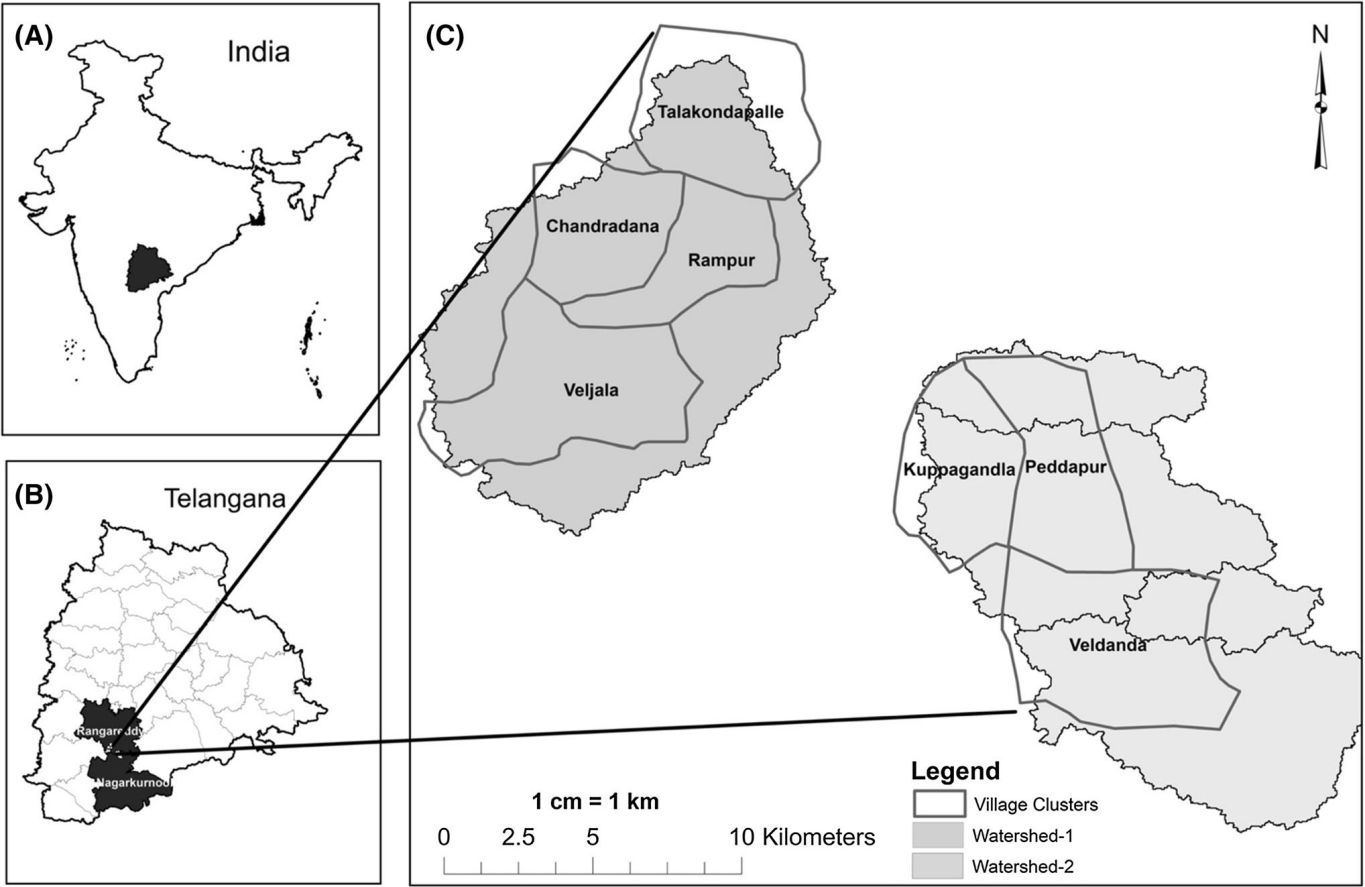
**a** Location map of the study region in India. **b** The study region (districts) within the state of Telangana. **c** The two watersheds within which the study villages are demarcated. Source: ISRO BHUVAN portal ( htpps://bhuvan.nrsc.gov.in/bhuvan_links.php, accessed 2016)

In this study, a watershed (WS) is considered as the unit of analysis as it is a part of a larger study that looks at the impact of transitions in farming systems on smallholder livelihoods and the environment. This paper is the first study in the series where the WS is not only considered as a hydrological unit but more as a social-ecological entity, which plays a crucial role in determining food, social, and economic security to rural people (Reddy and Syme 2015). We selected two WSs for the study to understand if the transitions were uniform or if substantial variation existed. WS-1 is located in Talakondapally Mandal (the smallest administrative unit within a district), covering four villages. WS -2 is in Veldanda Mandal, covering three villages. These Mandals are located in the Rangareddy and Nagarkurnool districts of Telangana State (Fig. 1). The total geographic area of WS-1 is 14 120 hectares (ha), and the boundary of the villages under study covered 9463 ha. WS-2 spans 13 694 ha, with 7701 ha falling within the study village boundary. Hence, for secondary data sources, we considered boundaries of the villages as the secondary data were aligned more with administrative boundaries than with hydrological ones.

Both watersheds fall in the Deccan Plateau (Telangana) and Eastern Ghat agro-ecological sub-region (AESR) 7.2. This sub-region is broadly characterized by deep loamy and clayey mixed red and black soils, with medium to very high available water capacity, and a growing season duration of 120–150 days. The climate is hot and moist in summer and mild and dry in winters, with an aridity index of 0.2 ≤ AI < 0.5 (Rao et al. 2019). Hence, it is classified as a semi-arid region. The districts are drought-prone, with an annual rainfall of 500–700 mm, which follows a seasonal pattern (Gajbhiye and Mandal 2000).

### Data collection

To achieve the aim of this research, we collected data using multiple methods sequentially to build a comprehensive picture of the transitions and their effects on farming HHs in the region. First, we started with a HH survey to obtain an overview of HHs in the region. The HH survey was conducted in both watersheds covering a sample of 3006 HHs. Surveys were face-to-face meetings with the HH head and were performed using a survey format. The survey provided an overview of information about the population, farm sizes, and categories i.e., large farmers (> 4 ha), medium farmers (2–4 ha), small farmers (1–2 ha), and marginal farmers (up to 1 ha); types of livestock; and different caste groups. The caste system in India is a social hierarchical system that has its origins in ancient India. This system, however, has been transforming since medieval times to several social reforms in modern India today (de Zwart 2000; Bayly 2001). Although several laws exist, stratification continues to exist in various forms. In modern India, the various castes are categorized into 4 main groups, i.e., forward caste (FC), backward caste (BC), scheduled caste (SC), and scheduled tribes (ST), which were also captured through the survey.

Second, using the above data, seven focused group discussions (FGDs) were organized. The objective of the first FGD was to obtain qualitative information on the overall narrative of how transitions occurred in the region pre-1997 to 2015, along with the drivers of change and its impact on HHs in the region. To achieve this, we selected HHs that had been in the region for the past 30–40 years. This was done in consultation with the head of each village council of all study villages. A village council in India is known as a *Gram Panchayat,* a local self-governance unit. From the list of potential participant HHs, 35 HHs were randomly selected and invited for the FGD. Further, we ensured that all farmer categories and caste groups, along with a representation of members of different age categories, i.e., old (> 60 years old), middle-aged (40–45 years old), and young (< 25 years old) were present. If the representation of one of these groups/categories was lacking, we substituted a randomly selected HH from the overrepresented group. This FGD was a mixed-gender group, with a total of 37 participants, of whom only four were women.

This was followed up by organizing the next five FGDs with HHs belonging to different farming system typologies in the region, identified through the HH survey data (Kuchimanchi et al. unpubl. results). The aim of these FGDs was similar to the first FGD in terms of mapping transitions and understanding their characteristics but with specific reference to the particular farming system. The five farming system typologies were crop without livestock (CWL), crop with dairy CD), landless with livestock (LWL), crop with small ruminants (CSR), and crop with diverse livestock (CWDL). Within each farming system typology, 30 HHs were randomly selected. Adequate representation from all farm size categories and social groups was ensured. These FGDs were again mixed-gender groups, and the actual participation varied from 25 to 30 members per FGD*.*

Although women participated in all six FGDs, an additional FGD was organized exclusively with women. Owing to socio-cultural reasons, women in India tend to participate in low numbers or do not voice their opinions in mixed-gender meetings. The objective of this FGD was to get a deeper understanding of how transitions affected women with specific reference to farming systems and practices, as transitions in farming systems could have different impacts on both genders. This FGD was organized as part of a monthly women’s self-help group meeting in one of the villages, as women of all age groups, castes, and farmer categories are usually present at such meetings. A total of 46 women participated in the FGD.

All seven FGDs lasted for 3–4 h, and the discussions were conducted in the local language, *Telugu*. For all FGDs, a participatory timeline-mapping tool (Hekkert and Negro 2009) supported with a list of questions to guide the discussions was used to achieve the objective of each FGD. The key questions in the FGDs were about major changes in farming systems, crops cultivated, livestock reared, fertilizer usage, livestock products, animal health, fodder resources, land use, and water resources. For the five FGDs specific to farming system typologies, questions related to the characteristics of farming systems and changes within each system were discussed. Concerning the impact on caste groups and women, along with the above, additional questions on aspects related to access to resources and challenges faced due to changes in farming systems were asked.

Considering the diversity in social status, farmer categories, caste, and gender in the FGDs, an experienced facilitator was present to moderate the discussions. The facilitator helped avoid domination by the wealthy, elderly, or socially forward groups and provide adequate time to document information in detail. All the discussions were documented on chart papers to maintain transparency and enhance interaction with the participants. As no major differences in narratives were perceived among FGDs, the documented information from all FGDs was summarized into a macro-picture of how transitions took place in the region, highlighting major aspects across a timeline as illustrated in Table 2. Further, the specific impacts on caste groups and women have also been highlighted in Table 2, in the results section as appropriate and described separately in the subsequent section.

Third, and lastly, to contrast and triangulate the information from the HH survey data and FGDs, we collected secondary government data from both local department offices and online government websites and land use land cover (LULC) evolution in the. The various government data sources consisted of population census 2001, 2011, crop statistics at the sub-district level between 1996 and 2015, the Agriculture at a Glance-Telangana state report -2018, statistics from the Agricultural and Processed Food Products Export Development Authority (APEDA), 2019, and livestock census data for 2007, 2012, the Basic Animal Husbandry Statistics, 2019, and the reports of Central Ground Water Boards published between 1997 and 2020.

For the LULC evolution analysis, land classes identified and mapped in the study area were according to the National Remote Sensing Agency (NRSA 2006) as defined below in brief:

- Settlement area: An area of human habitation that has a cover of buildings and basic infrastructure.
- Cropland-irrigated: Cropland under irrigation or lands that are cropped for two or more seasons in a year, as is often associated with irrigation.
- Cropland-rainfed: Cropland associated with rainfed crops under dryland farming with no irrigation (synonymous with areas with the cropping season-extending between June and October).
- Fallow land: Lands that are cultivated temporarily or kept uncropped for one or more seasons but not less than one year.
- Wasteland: Degraded or underutilized land that is deteriorating for lack of appropriate water and soil management but where key functions can be restored.
- Plantations: Areas under tree crops (agricultural/non-agricultural) planted adopting certain management techniques.
- Water bodies: Areas with surface water, e.g., ponds, lakes, and reservoirs or flowing as streams, rivers, canals, etc.

Agricultural crops in India are grown throughout the year in two main seasons. The LULC maps were combined for both seasons in an annual LULC map for the years 1997, 2005, and 2015. For this, only the total geographic area falling within the village boundaries within both watersheds was considered. Before processing the satellite imagery, a ground-truthing exercise was performed to identify samples of different land classes present in the villages using the global positioning system. Details of the data sources for the satellite imagery used are provided in Table 1.

**Table 1:** Data sources of satellite imagery used for the LULC study

Study year	Season	Acquisition date	Sensor	Path row	Resolution (m)
1997	Kharif-monsoon	05 October 1997	IRS 1C—LISS III—National Remote Sensing Centre, Hyderabad	100/69	30
	Rab-winter	07 & 20th February 1997			
	Zaid-summer	April 1997			
2005	Kharif-monsoon	01 September 2005	LANDSAT- Thematic Mapper (TM)–from USGS^a^	144/48	30
	Rabi-winter	17 November 2005			
	Zaid-summer	02 March 2005			
2015	Kharif-monsoon	12 October 2015	LANDSAT Satellite image Operational Land Imager (OLI) from USGS^a^	144/48	30
	Rabi-winter	17 December 2015			
	Zaid-summer	03 April 2015			

^a^http://glcf.umd.edu/data/landsat/

### Calculations and statistical analyses

We performed statistical tests to understand the impact of transitions on certain parameters (i.e., land and herd size) across caste groups and between watersheds. The statistical analyses were performed using the statistical program GenStat (GenStat Committee 2000) using the HH survey data. First, to compare land sizes and herd sizes of HHs between the watersheds, we used the Mann–Whitney U test because the data were not normally distributed. In these cases, the median and 25th and 75th percentiles are reported. The effect of the watershed and caste of the local communities on the variables land size and herd size was analyzed using the generalized linear model procedure, by the model:

$$Y_{ij}=\mu+\alpha_{i}+\beta_{j}=\alpha_{i\times j}+\epsilon_{ij},$$

where Y_ij_ is land or herd size per HH, _i_ is explained by the mean (μ); watershed i (α_i_) and caste j (β_j_) are the fixed factors; and (α_i_ × β_j_) is the interaction between watershed × caste, and (ε_ij_) is the residual error.

Pairwise post hoc comparisons between treatment means were done using Fisher’s least significant difference method. Dependent variables showed a skewed distribution and were converted to their natural logarithm. To ensure transformation of values of 0 into natural logarithm, we added one unit to all values. Once the tests were run, the mean values and confidence intervals were then back-transformed (Johnson et al. 1994) and subtracted by one unit. Herd size was expressed in tropical livestock units (TLU). The conversion factors were cattle = 0.7 TLU, buffalo = 1.5 TLU, sheep/goats = 0.1 TLU, and poultry = 0.01 TLU.

## Results

### Transitions in farming systems from 1997 to 2015

The transition in farming systems described here is a macro-picture of how transitions have occurred in the study region from 1997 to 2015 (Table 2) also with significant events as collected from the HH survey and the various FGDs conducted.

**Table 2:** Overall narrative of transitions in farming systems from before 1997 to 2015 in the study region

Timeline	Farming systems	Crops	Fertilizers	Livestock	Livestock products	Animal health	Fodder resources	Land use		Water resources
Before 1997	Rainfed mixed crop-livestock farming system was the most prevalent Livestock rearing was grazing-based Farming was mostly subsistence-oriented	Food crops, e.g., castor, sunflower, pearl millet, and sorghum, dominated	Manure was abundant and was used more than inorganic inputs for crop production	All households kept livestock, and different native breeds existed Large ruminants were mainly kept by forward castes, while lower caste groups reared small livestock Role of cattle to produce bullocks for draught purposes, declined	Wide range of livestock products existed and sold at village markets	Disease incidence was low Use of traditional medicine was prevalent	Common property resources and crop residues were abundant		More common property resources, less land under crop production	Natural water bodies and open wells were the main source. After 1995, borewells emerged due to village electrification
1997–2000	Irrigated crop production with borewells started Farming become more market orientated	New crops and fodder varieties introduced	Subsidies for inorganic crop inputs introduced	Flock sizes of sheep started to reduce specifically impacting the BC caste group	Decline in milk products (e.g., curd, *khoa*, ghee) and wool Focus on milk and meat production started		Grazing restrictions in forests, affecting poor and lower caste groups that reared livestock	Wastelands converted to crop lands benefitting lower caste groups in terms of gaining land for crop production Common property resources start to reduce–impacting all caste groups but more specifically poorer HHs and women who reared small ruminants and indigenous cattle		Borewell water available at 18–30 m, which became the main source of irrigation. Open wells declined
2001–2006	Farming systems became more specialized, with single species of livestock inclusive of all caste groups Farming systems with no livestock started to emerge	Shift to water-intensive cash crops, e.g., cotton, maize, groundnut, and green fodder crops; reduction in food crops Mechanization in crop production initiated	Reduction in farmyard manure due to reduction in indigenous cattle per household led to a gradual increase in the use of inorganic fertilizers	Improved and exotic livestock breeds introduced Increasing trend in milch cattle among all caste groups Livestock services (fuel and draught) replaced by cooking gas and vehicles Goat rearing turned into a seasonal activity for all poor HHs irrespective of caste Sheep and goats being raised on leased lands Improved and exotic livestock breeds, e.g., Holstein–Friesian and Murrah, replacing indigenous cattle and buffaloes Native poultry reduced; commercial poultry farms increased	Focus on liquid milk and sale of live small ruminants increased	New breeds introduced with higher disease incidence Higher production costs due to the use of modern medicine More healthcare and insurance available for larger ruminants	Cultivated fodder, post-harvest crop lands, and crop residues became sources of fodder	Increased land under crop production, which continues to expand		Decline in water resources began in both surface and groundwater
2007–2015	Shift to specialized livestock farming and semi-intensive system with high market orientation among all caste groups who had access to water	High focus on cash crops and vegetable and fruit production	Shortages of manure due to decrease in livestock per HH. Use of inorganic fertilizers increased		Establishment of government and private dairies increased New markets for exotic/crossbred cattle and small ruminants emerged		Increased use of external feed for cattle Leasing lands for grazing started	Lands converted to other uses, e.g., real estate, increased settlement area, plantations		80% Natural water bodies disappeared. Borewell depth increased to 180–250 m. Leasing of borewells by livestock keepers for water started

*Source: Compiled from information obtained from seven FGDs with different farmer categories and caste groups conducted in the year 2015. HHs–households*

Participants in the first FGD shared that transitions in farming systems started gradually since 1997, with major shifts occurring after the year 2000. Subsistence mixed farming systems were predominant before 1997, and almost all HHs had livestock then. Crop production was mostly rainfed, while livestock farming was grazing-based. Poultry keeping was mainly with indigenous scavenging birds, and every HH had a few. Farming was subsistence-oriented, and only surplus products were sold at local markets. Irrigated crop farming began in the late 1990s when village electrification and borewell technology emerged. Irrigated crops such as cotton, maize, groundnut, vegetables, fruits, and fodder crops like Napier grass (*Pennisetum purpureum*) and fodder sorghum replaced rainfed food crops such as castor, sunflower, pearl millet, and native variety of sorghum. These trends could be corroborated by crop production data provided by the agriculture department at the sub-district level for WS-2 (data for WS-1 were not available) for the period of 1996–2015. Major changes were seen in a few crops, e.g., the cropped area under sorghum dropped from 1081 ha in 1996–1997 to 20 ha by 2014–2015. The cultivation of pearl millet was around 103 ha, which completely disappeared by 2015. Similar trends were found for cotton and maize. In 1996, the area under cotton was 186 ha and no maize was cultivated. By 2015, the area under cotton had increased to 1548 ha and 419 ha, respectively. Vegetable cultivation also increased from just 30 ha in 1996 to 245 ha by 2015. The area under fodder sorghum increased from 3 to 38 ha in the same period.

In the same FGD, participants further shared that keeping livestock in the pre-1997^1^ period had multiple purposes such as providing food, manure, fuel, and draught power. They mentioned that ownership of good cattle breeds (identified as *Ongle, Deoni, Red Sindhi*, and *Krishna Valley*) was linked to having resources in terms of land, water, and finances. Large ruminants were predominantly owned by FCs, while lower caste groups reared small ruminants and poultry. As the mechanization of crop production and motorization of transport increased, the importance of bullocks decreased. Consequently, keeping reproductive cattle to produce bullocks reduced. To this, FGD participants from the CD system added that exotic dairy cattle breeds such as Jersey and Holstein–Friesian started to replace indigenous cattle breeds in 2004 and 2010, respectively. Similar was the case with buffaloes; indigenous buffaloes were replaced by *Murrah* buffaloes since 2002. This trend described in the FGDs was consistent with the government livestock census reports of 2007 and 2012 at WS level as it indicated a 28% increase in exotic/crossbred cattle and a decrease in indigenous cattle and buffaloes by 40% and 38%, respectively. The participants further added that while changes in breeds increased production and subsequently income, disease incidence in livestock is a drawback. Before 1997, animal disease incidence was low, and the diseases were easily cured with traditional medicines. However, with the introduction of exotic breeds, new diseases were reported, and traditional medicines were no longer useful. While animal health services are present, access to these services was reported to be better for large ruminants than for small ruminants. Insurance schemes are also in place but not considered functional by FGD participants due to low accessibility and laborious processing procedures.

In the case of small ruminants, FGD participants from the CSR system mentioned that changes took place both in terms of flock sizes and rearing systems since 1997. Traditionally, sheep were reared by HHs belonging to a livestock-keeping community called *Gollas* in the state, who are classified as BCs. Sheep rearers in the FGD reported that flock sizes have reduced from 5000 animals per HH in the past to 100–300 animals per HH. The adjustment in flock sizes was dependent on the availability of grazing lands and labor per HH, both of which have reduced over the years. Hence, more HHs keep sheep now than in the past, albeit in smaller flocks. Sheep migration has also stopped and is resorted to only under severe drought situations. *Deccani* was the dominant sheep breed pre-1997. This breed has been replaced with the *Red Nellore* sheep breed from coastal regions since 2000. Sheep farmers indicated that they prefer *Red Nellore* over *Deccani* as the former gains weight faster despite fodder scarcity. Participants shared that goats were reared by all caste groups but predominantly by women, poor and landless HHs. The breeds reared were native breeds, of which one was extinct, and could not be identified due to a limited database of local breeds in India. In the past, goat rearing was described as a year-round activity by many HHs. It is now a need-based activity for HHs, often done during the summer season or to cope with crop loss, loan repayment, or a sudden need for money. Government livestock census reports from 2007 to 2012 also report a drop-in sheep population (-41%), which could be related to dwindling flock sizes over time, while goat population shows an increase (26%) as it has turned into a seasonal activity for many HHs. While a general decrease in livestock population is seen at the HH level in the region, whether this change increased the economic value per unit is to be researched upon.

Participants in all FGDs indicated that indigenous poultry was kept by all HHs in the past and was an important source of food and income security for the poor, landless, and women. This trend is also indicated by the livestock census where native poultry rearing showed a drop by 82% between 2007 and 2012.

Further, the trends in both crop and livestock production described in all the FGDs do not seem to be limited to the study region but are seen across the state of Telangana. The Basic Animal Husbandry Statistics of 2019 indicate that livestock population changes in the region are similar to trends at the state level except for sheep and goats that show a substantial increase. This could be due to (i) lack of data for the study area before 2007 and (ii) small ruminant populations being linked to the presence of certain caste groups or influenced by HH needs. Similarly concerning the state’s cropping patterns, the states’ Agriculture at a Glance report indicates a trend towards the cultivation of non-food crops, as the area under food crops came down from 3.39 to 2.62 million ha between 2001 and 2016.

Further, participants from different farming systems FGDs reported a change in the kind of livestock products being sold, as the demand for raw milk increased. For instance, traditional farm-processed products like curd, buttermilk, *khoa* (thickened condensed milk), and ghee are not sold by HHs in local markets anymore. This role has been taken over by the government and private dairy units, to which the HHs now supply only raw milk. Moreover, dairy farmers shared that they prefer cows to buffaloes, owing to the better reproductive performance of the former (shorter inter-calving periods), which eventually results in higher income per year. Similar was the case with small ruminants and associated products, particularly for sheep. The demand for wool and other co-products diminished, and sales are currently limited to live animal sale for meat. These trends are aligned with state government data and APEDA, which show a nominal increase in wool production between 2001 and 2015 (i.e., 3.02 to 4.56 million kgs), while the state of Telangana (erstwhile Andhra Pradesh, before 2014) ranks first in sheep production nationally since 2008.

Lastly, Table 3 reports changes in LULC from 1997 to 2015, which further triangulate data from the FGDs. LULC changes indicated an increase in irrigated and rainfed cropland area by 734 ha and 3693 ha, respectively, mainly at the expense of wastelands, which decreased by 5330 ha. This could be due to an increase in population in the region, as the settlement area has increased from 36 to 475 ha between 1997 and 2015. Further, the reduction in wastelands that were used for grazing livestock resulted in reduced fodder availability for many HHs rearing livestock. This situation worsened further around the year 2002, and grazing restrictions were also levied on nearby forest areas.

**Table 3:** Changes in land use and land cover from 1997 to 2015 in both watersheds combined

Land classification	LULC 1997 area (ha)	LULC 2005 area (ha)	LULC 2015 area (ha)
Settlement area	36	253	475
Crop land: irrigated	999	2427	1733
Crop land: rainfed	8807	7841	12 500
Plantations	52	612	612
Waste land	7093	5925	1763
Surface waterbodies	177	105	80
	17 164	17 164	17 164

Source: Satellite imagery from National Remote Sensing Centre -1997 & LANDSAT -2005 and 2015 (refer to Table1); LULC area is the area within the village boundaries in both watersheds

Reduction in access to grazing resources along with a sharp increase in cropland, both irrigated (ca. 75%) and rainfed (ca. 40%), not only impacted the availability of fodder for livestock but also livestock rearing in general. Participants from the farming system FGDs shared that currently almost half of the HHs in the villages do not own livestock, also indicated by the HHs survey as 48%. The FGD participants in the CD system stated that dairy producers managed this situation by cultivating perennial fodder crops due to fodder seed subsidies provided by the government and dairy cooperatives. Hence, grazing-based cattle systems eventually changed into semi-stallfed systems, with some grazing on fallow croplands or wastelands if available.

Based on information shared by participants from the crop with small ruminant farming system, it is indicated that small ruminant farming transformed into a modern grazing-based system. Earlier, small ruminants were raised entirely on village common lands or wastelands, with surface water bodies as water sources. Now, cropland, orchards, private lands, and borewells for water are leased to rear small ruminants. According to the participants, sheep rearers could find lands to graze their animals more easily than goat rearers, as goats are browsers, and require lands with tree cover. Goat keepers stated that the availability of wastelands with tree cover has decreased considerably. Hence, goats are now reared in small flocks in seasons when crop farming is low or absent or as per need, rather than as a year-round activity as in the past. Small ruminant rearers added that only HHs that could invest in leasing lands and borewells now continue small ruminant rearing with large flocks as a full-time occupation. The traditional barter systems between small ruminant farmers and crop farmers, where crop residues were bartered against manure, no longer exist.

The participants from all FGDs also stated that while these changes in crop and livestock production took place, water scarcity in the region has also increased. Before 2010, borewells were 18–30 m deep. However, currently, borewells yield water only at 180–250 m depth. Natural surface water bodies have also disappeared, affecting small ruminant keepers the most, as they now have to invest in buying water for livestock. Both these findings can be corroborated by data from the Central Ground Water Board (2019), as the region has moved from semi-critical to critical status in 2013–2017, indicating the overuse of groundwater. Similarly, the LULC study also indicates that water bodies have reduced by 79% in the region (Table 3).

### Impact of transitions on caste groups

**Fig. 2:**
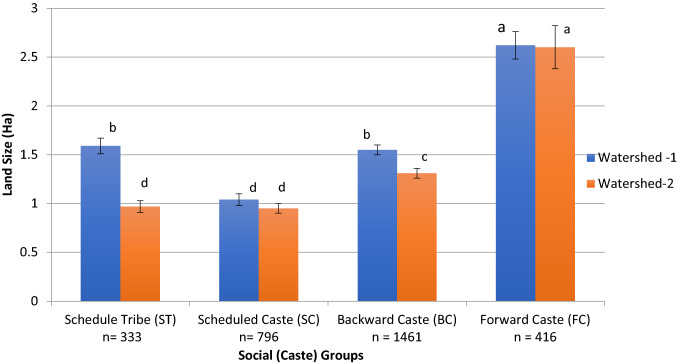
Land size (in ha) across caste groups (Scheduled tribe, ST; Scheduled caste, SC; Backward caste, BC; and Forward caste, FC) in both watersheds. Source: HHs survey, 2015

**Fig. 3:**
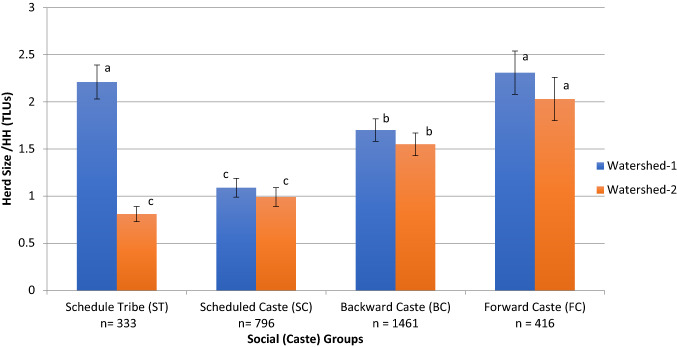
Herd size (in TLUs) across caste groups (Scheduled tribe, ST; Scheduled caste, SC; Backward caste, BC; and Forward caste, FC) in both watersheds. Source: HHs survey, 2015

In continuation to the above section, we further analyze the impact of transitions on different caste groups in the study region, particularly to gain insight into differences in land and herd size between castes and watersheds. Figures 2 and 3 present the land and herd sizes of the different caste groups. We found a significant interaction between watersheds and caste groups, which can be mainly explained by the differences observed between watersheds for the ST caste group. We found that the FC communities had the highest land size per HH (2.6 ha) in both watersheds, whereas SC communities in both watersheds had the smallest land sizes (average of 1.0 ha/HH). The ST and BC communities had higher land sizes in WS-1 than in WS-2. Similar was the case with herd size, where the FC communities had the highest herd size per HH, except for the STs in WS-1 (average of 2.3 TLUs/HH), followed by the rest. These results align with the information from the FGDs discussed above, wherein land and herd sizes generally still followed the caste hierarchy. However, a change in ownership patterns for large ruminants is observed, in contrast to the past, where lower castes also own dairy cattle.

### Impact of transitions on women

According to the women participants, who attended all FGDs, there were improvements since 2000 regarding access to livestock, livestock ownership, and decision-making associated with livestock rearing had increased. This was attributed to the increased participation of women in government-initiated self-help groups in their villages. According to them, participation in self-help groups helped women to gain access to new technologies and own livestock. However, they also reported an increased workload regarding farming and responsibility in terms of loan repayments. With respect to the increase in workload, women expressed that rearing improved cattle in stallfed systems demanded more time, e.g., for feeding, cleaning sheds, and animal health care, which was limited when compared to rearing cattle in grazing-based systems. Similar sensitivities were shared by women regarding changes in crop production. For instance, the shift from rainfed food crops to irrigated cash and vegetable crops also increases the workload particularly for tasks from multiple harvests to packing, which is carried out exclusively by women.

With regard to the rearing of small livestock, women shared that the rearing of goats and poultry by them has particularly decreased compared to the past. According to them, reduced access to grazing lands and tree cover in the region due to land use changes (Table 3) meant longer grazing hours and hence was avoided by older women and women with young children. Meanwhile, the reduction in native poultry rearing was due to developments in settlement areas and closer proximity of houses within the settlement areas, which led to reduced scavenging areas for chickens and conflicts among HHs.

## Discussion

### Characteristics of transition

Our study aimed at describing transitions in farming systems at a regional scale and analyzing their impacts on livestock rearing and smallholder livelihoods, along with insights on caste groups and women. The findings from the FGDs, HH survey data, LULC data (Table 3), and statistical tests (Figs. 2 & 3) all indicated that trends of how the transitions in farming systems occurred in both watersheds were similar. They were completely in the direction of market orientation and happened in a relatively short period. Matthei and Smith (2008) and Butler et al. (2014) show that such transitions are possible despite the diversity of social groups within a region. Here, community aspirations to improve living standards tend to overcome the social and cultural identities bringing in flexibility to adapt to changing circumstances. Farming systems before 1997 were mostly subsistence-oriented, with mixed crop-livestock production and livestock having diverse functions (Ali 2007; Kumar and Singh 2008). Between 1997 and 2015, the subsistence farming system disappeared, and specialized and market-oriented production systems emerged. The multiple roles of livestock in mixed farming systems reduced to the role of food production mainly. In these new systems, the investments, cost of production, and input use have become relatively high, e.g., inputs for cultivating cash crops, leasing land for grazing livestock or growing fodder, leasing or drilling borewells for water, farm mechanization, purchase of feed, and animal healthcare (Singh et al. 2014; Gathorne-Hardy 2016; Ghosh et al. 2017; Kuchimanchi et al. unpubl. results). Further, these changes do not seem to be limited to the study region as similar trends in changes in agricultural landscapes, livestock holdings, and cropping patterns are reported in Telangana (Reddy et al. 2016) and across India (Government of India 1997, 2002, 2007, 2012; Amjath-Babu and Kaechele 2015; Behera et al. 2016). Such a relatively fast and region-wide transition from subsistence farming to market-oriented farming has also been reported in Africa, Latin America, and Asia by Reardon et al. (2019).

### Drivers of transition

The transitions in farming systems from 1997 to 2015 in the two watersheds were driven by technological interventions, development programs promoting green and white revolution technologies with integrated approaches, and increased market demand for cash crops and certain livestock products (Behera et al. 2016; Gathorne-Hardy 2016). An important technological intervention that triggered the transition process is village electrification, which prompted the use of motor pumps for extracting water from borewells, thereby facilitating water-intensive crop and livestock production (Tian et al. 2014). Further, we find that the sudden increase in water availability in dryland regions, due to the development programs with integrated approaches, e.g., watershed development, seemed to be a lucrative incentive for smallholders to adopt new technologies and diversify faster (van Ginkel et al. 2013) facilitating rapid transitions in farming systems.

The major market for the study region is Hyderabad, one of the biggest cities in India, growing from 3.6 million inhabitants in 2001 to 11.5 million in 2018. While the population growth in itself was an important reason for the increased demand, the income growth of the urban population also adds to this by influencing changes in food consumption patterns (Oosting et al. 2014; Kumar et al. 2017; Van der Lee et al. 2018; Reardon et al. 2019). Hyderabad has the highest food consumption expenditure per month in Telangana, of which the highest share comprises animal products (32% of the total) (Kumar et al. 2017), indicating a huge and possibly growing demand in this sector.

While FGDs identified several drivers that triggered the transitions, this is not an exhaustive list. Other drivers might have also played an important role, such as the influence of external policy situation, input of remittances, or differences in education and knowledge gains between castes or gender (Thompson et al. 2007; Reardon et al. 2019). The contribution of these other aspects to farming systems transitions needs further study.

### Impacts of the transitions in farming systems on smallholder livelihoods

While transitions in farming systems across India and the study watersheds might be beneficial in some ways, however, not all is positive (George 1986, 2014; Pingali 2012; Hinz et al. 2020). Programs with integrated approaches, e.g., WDPs make development dynamic and involve trade-offs as well (van Ginkel et al. 2013). For example, the transitions in the study region favored the expansion of croplands, increased use of green revolution technologies, and more focus on milk production. It also reduced the production of other livestock products, reduced diversity within farming systems, and eroded animal genetic diversity. This is a trend generally reported in the literature in transitions from subsistence to market-oriented farming systems (Puskur et al. 2004; Jayne et al. 2014; van Ginkel et al. 2013; Oosting et al. 2014; Gathorne-Hardy 2016).

Further, increased production by some farmers in the study area has triggered regional changes in water, land, and labor scarcity for others, making it compulsory for all to intensify production. The transition, therefore, was not a free process but a compulsory adaptation, inclusive of social and cultural differences due to changing circumstances (Matthei and Smith 2008). This can be inferred because the HHs without agricultural activities or with the traditional subsistence mixed farming system together is around 10% in both watersheds. This implies that once the transition process gained momentum, farmers could either join in or step out from agriculture, and is in line with Dorward et al. (2009) and Reardon et al. (2019). An additional marginalization was also witnessed in our study watersheds: we observed that only a limited fraction (38%) of HHs could maintain both crop and dairy cattle, while half of the HHs (48%) did not rear livestock owing to inadequate water resources, which is already a problem in dryland regions. This dramatic change further implies that the majority of the HHs are susceptible to risk due to the lack of diversification at the HH level, particularly the absence of livestock. The lack of crop-livestock integration may also have negative implications on agricultural production and revenue in the long term (Kuchimanchi et al. unpubl. results). Increasing water scarcity in the region as reported by the respondents is also in line with Sishodia et al. (2016) and the Central Ground Water Board’s report (2017), which indicates a decrease in groundwater levels both within the study region and across the state.

Despite the considerable increase in cropland area, land size per HH has likely reduced over time due to fragmentation of land, e.g., by the division of property among siblings, as both settlement area and HH population (Government of India 2001, 2011) in the watersheds show an increase by 12.2% and 16%, respectively. This trend seems to be across the state; the agricultural statistics report of Telangana (2016) shows that the average landholding in the state in 2010–11 was 1.12 ha against the all-India average of 1.16 ha. Hence, it is likely that many HHs in the study region have become marginalized during the transition process and have migrated, changed their occupations, or become wage laborers.

### Effect on caste groups and women

Many of the approaches of the green and white revolutions are still being out-scaled through development policies and programs as a means of poverty alleviation, e.g., integrated WDPs, self-help group movements, or agricultural subsidies and schemes. In this context, the transitions in the two watersheds showed that lower caste groups now own improved cattle (Figs. 2 and 3) and have consequently moved up the livestock ladder (Udo et al. 2011). We also found some exceptions where the STs in WS-1 had both land and herd sizes as high as those of the FCs. These changes among lower caste groups can be attributed to several government-sponsored schemes (Reddy et al. 2016) which are specifically designed for their upliftment (Government of India 2008). Nevertheless, our study shows that FCs continue to own the largest land sizes and cattle herd sizes, as in the past.

The transition towards intensification and market orientation was women-inclusive, as women had increased access to technologies, information, and livestock resources. However, a perceived increase in workload for women was reported in our study which in line with other studies in India (Vepa 2005; van Ginkel et al. 2013; Pattnaik et al. 2017). In this case, this was in the form of the shift from grazing-based livestock rearing to stallfed market-oriented systems (Köhler-Rollefson 2012) and from rainfed food crops to irrigated cash and vegetable crops. This perception of increased workload existed as certain activities in crop-livestock production are predominantly done by women, along with the already existing traditional roles within the home (Lastarria-Cornhiel and Bank 2008). Furthermore, a general reduction in small livestock rearing and poultry rearing by women is seen, depriving them of potential activities to gain financial and nutritional security (Conroy et al. 2005; Chatterjee and Rajkumar 2015).

## Conclusions

Studying transitions in farming systems at a regional level highlighted various interactions in the study region, i.e., between diverse farming systems, between farming system development and natural resource use, and between regional transition and different social groups. We demonstrate how these elements impacted the development trajectory of a region with a dual effect of both enhanced incomes and marginalization of some farming HHs therein.

We found that the regional transitions in farming systems have occurred in a short period, and subsistence mixed farming systems have almost completely transformed into market-orientated specialized systems in the region. Further, the function of livestock in farming changed from a multi-purpose role in the past to a market-oriented food production role. The major drivers of the transitions were found to be technological interventions, development programs with integrated approaches, and market demand for certain agricultural produce. While the transitions led to increased production by some HHs, they also led to the scarcity of water, land, and labor for others. The transition, therefore, was not a free process but a compulsory adaptation, inclusive of social and cultural differences among the HHs in the region. The HHs had to either intensify production to adapt to the transforming prospects or get marginalized. The implications of these transitions were progressive in the case of lower caste groups, as they have moved up the livestock ladder and gained assets. However, in the case of women, it was perceived unfavorable in terms of increased workloads and reduced food and financial security. Our study, thus, provides deeper insights into how transitions impact multiple aspects of smallholder livelihoods. Finding from this study could contribute to the strengthening of rural development policies to reduce risks in agricultural production, e.g., water scarcity stemming from already operational programs.

## Footnotes

1 Livestock population trends pre-1997 at village level could not be triangulated with secondary data due to data gaps, hence trends from 2007 to 2012 only have been presented.
